# AI-enabled multi-omics integration in colorectal cancer: from molecular stratification to clinical translation

**DOI:** 10.3389/fcell.2026.1797221

**Published:** 2026-04-30

**Authors:** Honghua Su, Tiangui Wang, Chaofan Chen

**Affiliations:** 1 Department of General Surgery, Kunming Municipal Hospital of Traditional Chinese Medicine, The Third Affiliated Hospital of Yunnan University of Chinese Medicine, Kunming, China; 2 Department of Anorectal, Kunming Municipal Hospital of Traditional Chinese Medicine, The Third Affiliated Hospital of Yunnan University of Chinese Medicine, Kunming, China

**Keywords:** artificial intelligence, biomarker discovery, clinical decision support, colorectal cancer, machine learning, multi-omics, precision medicine

## Abstract

Colorectal cancer (CRC) remains a heterogeneous disease for which improved molecular stratification is needed across the clinical pathway. Multi-omics technologies have expanded insight into CRC biology, and artificial intelligence (AI) has created new possibilities for integrating molecular, pathological, imaging, and clinical data. This review examines how these approaches are being applied across screening, diagnosis, treatment, and prognosis, with particular emphasis on their clinical relevance and translational limitations. We argue that, despite encouraging advances in biomarker discovery and risk prediction, most current studies remain retrospective and are constrained by heterogeneity of data sources, limited standardisation, weak interpretability, and insufficient external or prospective validation. AI-enabled multi-omics integration has substantial potential in CRC, but meaningful clinical impact will require rigorous validation and implementation frameworks suited to routine care.

## 1 Introduction

Colorectal cancer (CRC) is a major global health challenge, representing nearly 10% of the 19.3 million cancer cases worldwide in 2020 (Sung et al., 2021). It is the third most common malignancy and the second leading cause of cancer-related mortality, with 3.2 million new cases and 1.6 million deaths projected by 2040 (Yoshino et al., 2021). CRC is a highly heterogeneous disease driven by complex genetic, epigenetic, and microenvironmental alterations, which substantially influence therapeutic response and clinical outcome (Punt et al., 2017; Tepeli et al., 2021). Although surgery, chemotherapy, and radiotherapy remain the cornerstones of CRC treatment and have improved survival in many patients (Biller and Schrag, 2021), their long-term benefits are limited for a considerable proportion of individuals, largely owing to interpatient molecular heterogeneity and treatment resistance (Punt et al., 2017; Andrei et al., 2022; Zhao et al., 2024). Together, these features make CRC a challenging disease to manage and highlight the need for more precise biologically informed approaches to classification and treatment (Punt et al., 2017; Buikhuisen et al., 2020).

Advances in high-throughput multi-omics technologies including genomics, transcriptomics, epigenomics, proteomics, metabolomics, microbiomics, and lipidomics have enabled systematic interrogation of CRC across multiple molecular layers in a spatiotemporal context (Tang et al., 2019). While genomic profiling has facilitated the identification of CRC subtypes (Zhao et al., 2022), DNA-level alterations do not necessarily translate into functional changes in gene expression or protein activity (Xu J. Y. et al., 2020). Transcriptomic analyses have led to the definition of consensus molecular subtypes (CMS) (Guinney et al., 2015), yet the relationship between mRNA, protein abundance, and treatment response remains incompletely understood (Xu J. Y. et al., 2020).

Integrated multi-omics analyses provide a holistic view of cancer biology and enable the identification of molecular networks that drive tumour initiation and progression (Karczewski and Snyder, 2018; Hasin et al., 2017). In particular, proteome-centric integration strategies, supported by mass spectrometry–based proteomics, offer critical insights into functional dysregulation beyond genomic alterations and hold promise for biomarker discovery and personalized therapy development (Xu J. Y. et al., 2020; Li S. et al., 2023; Liu et al., 2024; Zhang et al., 2025).

Artificial intelligence (AI), especially deep learning (DL), has emerged as a powerful tool for extracting clinically actionable information from high-dimensional, multimodal datasets (Esteva et al., 2019; Kann et al., 2021). By integrating imaging, genomic, and molecular data, AI-based approaches have shown utility across several CRC tasks, including real-time polyp detection, radiogenomic prediction of molecular features, pathology-based prognosis modelling, and multimodal risk stratification; however, reported performance and generalisability vary substantially across data modality, cohort composition, and validation setting (Kann et al., 2021).

**FIGURE 1:**
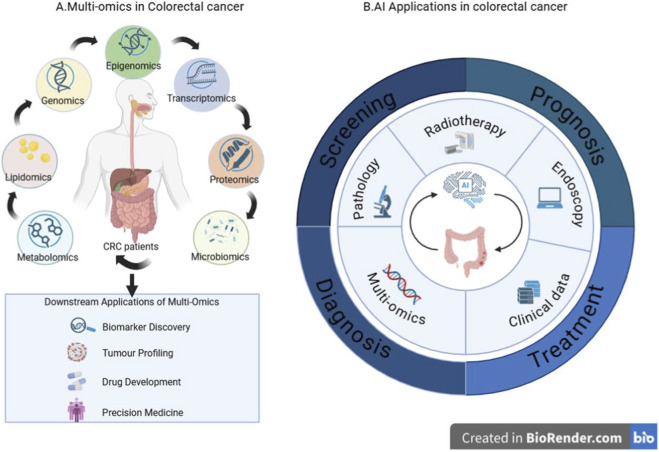
Overview of multi-omics profiling and AI applications in CRC. **(A)** Major omics layers involved in CRC, including genomics, epigenomics, transcriptomics, proteomics, metabolomics, lipidomics, and microbiomics, and their downstream applications in biomarker discovery, tumour profiling, drug development, and precision medicine. **(B)** AI–driven applications across the CRC care continuum, integrating clinical data, imaging, pathology, and multi-omics information to support screening, diagnosis, treatment, and prognosis.

Against this background, the present review is organised around the major omics layers relevant to CRC and their integration with AI across screening, diagnosis, treatment, and prognosis. The overall conceptual framework is outlined in Figures 1A,B.

This article was conducted as a structured narrative review. A targeted literature search was performed in PubMed to identify studies related to colorectal cancer, multi-omics technologies, and artificial intelligence. We prioritized recent and representative studies according to clinical relevance, methodological relevance, and their contribution to understanding AI applications and cross-omics integration in CRC. This review was not designed as a formal systematic review; therefore, no PRISMA-based study selection workflow was applied.

## 2 Multi-omics in CRC

### 2.1 Genomics of CRC

Genomic profiling has become a cornerstone in CRC management, enabling precise diagnosis and treatment. Key mutations, such as those in KRAS, BRAF, and PIK3CA, are crucial for understanding tumour biology and tailoring therapies. For instance, KRAS mutations occur in approximately 40% of CRC cases, leading to continuous activation of the RAS-MAPK pathway and resistance to anti-EGFR therapies like cetuximab and panitumumab (Ahmad et al., 2021; Vitiello et al., 2019). Similarly, BRAF V600E mutations, found in 8%–10% of patients, contribute to more aggressive disease and resistance to standard therapies. Targeted inhibitors of BRAF and MEK, such as vemurafenib, offer more personalized treatment options (Dankner et al., 2018; Sanchez et al., 2018). Furthermore, PIK3CA mutations, which activate the PI3K-AKT-mTOR pathway, are associated with resistance to chemotherapy and anti-EGFR therapies in 15%–20% of CRC patients (Hu et al., 2021; Rascio et al., 2021). These insights allow clinicians to optimize treatment choices and avoid ineffective therapies.

Epigenetic modifications, including DNA methylation and histone modifications, also play a crucial role in CRC development and progression. Altered methylation patterns, such as hypermethylation of tumour suppressor genes and hypomethylation of oncogenes, contribute to carcinogenesis and microsatellite instability (MSI) (Zamani et al., 2018; Berdasco and Esteller, 2019). Non-invasive biomarkers, including methylation in cfDNA and CTCs, hold potential for early CRC detection (Galanopoulos et al., 2017; Krishnamurthy et al., 2017). The role of 5-methylcytosine (5mC) and 5-hydroxymethylcytosine (5hmC) in CRC is increasingly recognised, with emerging studies suggesting their value in diagnostics and prognosis (Belkhiri and El-Rifai, 2014; Ichimura et al., 2015; Hur et al., 2014). These epigenetic markers, along with genomic alterations, provide important insights into CRC’s molecular landscape.

In CRC, genomic abnormalities such as single nucleotide variations (SNVs), copy number variations (CNVs), and microsatellite instability are prevalent. KRAS mutations are a major driver of CRC proliferation and metastasis (Zhang et al., 2009). Moreover, somatic CNVs detected in circulating tumour DNA have emerged as early biomarkers for CRC detection (Molparia et al., 2018). MSI, a key biomarker in CRC, also predicts the efficacy of PD-1 inhibitors in metastatic CRC patients, offering new therapeutic avenues (Lin et al., 2020). Together, these genomic and epigenetic insights are pivotal in refining CRC diagnosis, treatment, and prognosis, and in advancing precision medicine for this disease.

### 2.2 Transcriptomics of CRC

Transcriptomic analysis has proven essential in uncovering biomarkers within the CRC microenvironment, particularly by evaluating the composition of immune and stromal cell populations. Tumours of different molecular subtypes exhibit distinct tumour microenvironment (TME) characteristics, which correlate with tumour mutational burden, affecting prognosis and the efficacy of immunotherapies (Petitprez et al., 2018). Recent advancements, particularly single-cell RNA sequencing (scRNA-seq), have revealed insights into the involvement of neutrophils in iron metabolism and migration in CRC (Gao et al., 2022; Zhang Y. et al., 2020). This technology holds promise in providing more specific diagnostic and prognostic biomarkers, along with feasible therapeutic targets (Zhang et al., 2014). RNA-seq has also facilitated the identification of differentially expressed genes in CRC, enabling the development of risk score models for prognosis (Zhang X. et al., 2023). Pathways such as Wnt/β-catenin, PI3K/Akt, and TGF-β are crucial in CRC progression and serve as potential biomarkers (Sebio et al., 2014; Duan et al., 2018). Further, genes like c-MYC, KRAS, BRAF, and PIK3CA have been identified as key predictive markers for CRC prognosis (Marmol et al., 2017).

Despite its potential, transcriptomics in CRC research faces challenges. Current technologies have not yet achieved true single-cell resolution, and capturing the structural and functional diversity of transcripts, such as alternative splicing and modifications, remains a limitation. Moreover, the vast scale and complexity of transcriptomic data pose significant hurdles in data processing, integration, annotation, and interpretation. The development of more efficient algorithms and tools is critical to overcoming these challenges. While transcriptomics is pivotal for identifying biomarkers and assessing prognosis, its clinical applications face issues such as standardization, reproducibility, and sensitivity.

Genomic instability and epigenetic changes in CRC often lead to aberrant alterations in the transcriptome, reflecting the cell’s activity at any given time (Wang et al., 2009; Habermann et al., 2008). High-throughput RNA-Seq has enabled a more comprehensive mapping of the CRC transcriptome, revealing novel transcripts, alternative splicing events, and gene fusion products (Wang et al., 2009; Morozova et al., 2009). Studies using RNA-Seq to compare CRC tumour tissues with adjacent normal tissues have identified key activated pathways, including extracellular matrix remodeling and metabolic pathways, which are associated with tumour metastasis (Wu et al., 2012). In another study, the most frequently altered genes in CRC, including APC, TP53, and KRAS, were identified through high-throughput sequencing (Han et al., 2013). RNA-Seq is increasingly becoming the preferred method for profiling the CRC transcriptome, offering deeper insights into its genetic alterations (Wang et al., 2009).

### 2.3 Proteomics of CRC

Proteomics has emerged as a critical approach for identifying functional biomarkers in CRC, particularly proteins that can be detected in accessible biofluids such as blood, urine, and saliva (Tjalsma, 2010; Alvarez-Chaver et al., 2014). Compared with genomic or transcriptomic alterations, the proteome represents the functional output of the genome and is directly linked to tumour behaviour. Numerous studies have demonstrated that CRC is characterized by distinct proteomic signatures, including dysregulated expression and post-translational modifications (PTMs), which play essential roles in early diagnosis, prognostic stratification, and therapeutic intervention (Zhu et al., 2022). Recent advances combining mass spectrometry with machine learning (ML) have further enhanced biomarker discovery; for example, a convolutional neural network model identified a five-protein panel (SAA2, APCS, APOA4, F2, and AMBP) for early and advanced CRC detection (Ahn et al., 2019).

Proteomic profiling of tumour tissues and stromal compartments has revealed proteins involved in CRC initiation, progression, and invasion. Differential expression of ACTBL2, DPEP1, cyclophilin A, annexin A2, and aldolase A has been consistently reported across tissue-based proteomic studies using LC–MS platforms (Ghazanfar et al., 2017; Hao et al., 2017; Yamamoto et al., 2016). Proteomics has also enabled the identification of stromal and microenvironment-related biomarkers, including LTBP2, OLFML3, CDH11, CALU, and FSTL1, which are implicated in tumour migration and invasion (Torres et al., 2013). Moreover, proteome-level analyses of patient-derived organoids revealed that activation of MYC and enrichment of the TRiC chaperonin complex predicted sensitivity to palbociclib, highlighting the potential of proteomics for guiding treatment selection (Papaccio et al., 2023). Large-scale proteomic classification studies further identified biologically distinct CRC subtypes associated with immune regulation and therapeutic response, including resistance to radiotherapy and anti-EGFR therapy mediated by HSF1 and HDAC6, respectively (Li Y. et al., 2023).

Circulating proteomics has shown particular promise for non-invasive CRC screening, risk stratification, and disease monitoring. Multiple blood-based protein panels have demonstrated favorable diagnostic performance, with reported sensitivities exceeding 70% and specificities approaching 90% (Ivancic et al., 2020; Bhardwaj et al., 2019). Proteins such as EGFR, LRG1, ITIH4, HPX, SOD3, osteopontin, and amphiregulin have been repeatedly validated in plasma and serum cohorts (Ivancic et al., 2020; Bhardwaj et al., 2019; Chantaraamporn et al., 2020). Chemokines including CXCL8 have also shown superior diagnostic performance compared with carcinoembryonic antigen (CEA) (Paczek et al., 2020). Importantly, integration of blood proteomics with polygenic risk scores and clinical models has improved CRC risk stratification and informed individualized screening strategies (Sun et al., 2024).

In metastatic CRC, proteomic analyses of circulating tumour cells revealed heterogeneous epithelial–mesenchymal transition (EMT) states, identifying ERBB2, COL6A1, and CAVIN1 as promising metastasis-associated biomarkers and potential therapeutic targets (Huang et al., 2022). Despite these advances, effective integration of proteomics with other omics layers remains a major challenge, underscoring the need for standardized, scalable, and clinically translatable analytical frameworks.

### 2.4 Microbiomics of CRC

Microbiome profiling has identified CRC-associated microbial signatures with potential value in screening, diagnosis, and treatment-response prediction. Advances in 16S rRNA sequencing, shotgun metagenomics, and quantitative PCR have enabled increasingly detailed characterisation of gut microbial alterations in CRC (Liu et al., 2023; Holmes et al., 2012; Clos-Garcia et al., 2020; Zeller et al., 2014; Zackular et al., 2014; Yang et al., 2020).

Accumulating evidence indicates that the gut microbiome plays a critical role in CRC screening, diagnosis, and therapeutic response prediction. Specific microbial taxa, such as *Fusobacterium* nucleatum, are enriched in patients with adenomas and CRC and can be detected in fecal samples, highlighting their potential as non-invasive screening biomarkers (Guo et al., 2018; McQuade et al., 2019). Beyond local intestinal effects, recent studies have demonstrated that gut-derived microbial DNA can be detected in the circulation and may discriminate between different cancer types, supporting the concept of microbiome-based liquid biopsy approaches (Li et al., 2022). These findings underscore the systemic relevance of gut dysbiosis in CRC pathogenesis and clinical management.

Integration of microbiomics with metabolomics has further strengthened biomarker discovery in CRC. Microbial metabolites, including short-chain fatty acids, bile acids, and polyamines, have been consistently linked to colorectal carcinogenesis (Weir et al., 2013; Brown et al., 2016; Lin et al., 2019). For example, altered levels of acetate, butyrate, and secondary bile acids have been observed in CRC tissues and fecal samples, demonstrating diagnostic potential. Large-scale serum metabolomic analyses have identified reproducible metabolite signatures associated with gut microbiota that distinguish healthy individuals, adenoma patients, and CRC patients with high diagnostic accuracy (Chen et al., 2022). Collectively, these data suggest that microbiome–metabolome interactions are central to CRC development and represent promising targets for early detection, prevention, and personalized intervention strategies (Thomas et al., 2017; Nakatsu et al., 2015; Kwong et al., 2018).

### 2.5 Metabolomics of CRC

In CRC, metabolomics provides a functional readout of tumour-associated biochemical reprogramming and has become increasingly relevant for biomarker discovery using minimally invasive biospecimens such as blood, urine, stool, and saliva (Troisi et al., 2020; Zhang et al., 2015; Dunn et al., 2011; Ivanisevic and Want, 2019). Both targeted and untargeted metabolomic approaches have been applied to characterise metabolic alterations associated with CRC development and progression (Vignoli et al., 2019; Commisso et al., 2013).

Accumulating metabolomic studies across diverse CRC biospecimens have consistently demonstrated profound metabolic reprogramming during tumour initiation and progression (Wang et al., 2010; Nannini et al., 2020). Using analytical platforms such as nuclear magnetic resonance (NMR), gas chromatography–mass spectrometry (GC–MS), and liquid chromatography–mass spectrometry (LC–MS), investigators have identified dysregulation of pathways including glycolysis, the tricarboxylic acid cycle, amino acid metabolism, lipid metabolism, bile acid metabolism, and polyamine synthesis (Udo et al., 2020; Ning et al., 2021). Several studies have shown that metabolite profiles can distinguish healthy individuals, patients with benign polyps, and those with CRC, as well as differentiate early-stage from advanced-stage disease (Gu et al., 2019; Kim et al., 2019). Notably, polyamines (e.g., putrescine and cadaverine), short-chain fatty acids, bile acids, and sphingolipids have repeatedly been reported as candidate biomarkers for CRC diagnosis and prognosis (Clos-Garcia et al., 2020; Lin et al., 2019; Yang et al., 2019; Song et al., 2018).

At a functional level, the metabolome directly reflects biochemical reaction outputs and is highly responsive to both intrinsic oncogenic signaling and extrinsic environmental stimuli (Martinez-Reyes and Chandel, 2021; Wishart, 2019). Tumour cells undergo extensive metabolic rewiring to sustain uncontrolled proliferation, while immune cell differentiation and function are likewise governed by metabolic programs (Schmidt et al., 2021; Johnson et al., 2016; Boothby and Rickert, 2017). These metabolic vulnerabilities generate characteristic metabolic phenotypes that can be exploited for early cancer detection, disease monitoring, and therapeutic targeting (Schmidt et al., 2021). Recent clinical and preclinical studies have further highlighted the value of metabolomics in drug discovery and pharmacodynamic evaluation, including the identification of metabolite signatures associated with treatment response (Zhang et al., 2018; Yin et al., 2022). Nevertheless, no single omics layer sufficiently captures the complexity of CRC biology, underscoring the necessity of integrating metabolomics with other omics technologies to achieve robust biomarker validation and mechanistic insight.

### 2.6 Lipidomics of CRC

Lipidomic profiling has revealed clinically relevant metabolic perturbations in CRC, with mass spectrometry–based approaches enabling detailed characterisation of lipid signatures associated with tumour progression and prognosis (Burla et al., 2018; Wang R. et al., 2020; Kyle, 2020). These analyses provide insight into CRC-related lipid remodelling and may support biomarker discovery and risk stratification (Wang R. et al., 2020; Kyle, 2020).

In CRC, growing evidence indicates that dysregulated lipid metabolism plays a critical role in tumour initiation and progression. Recent studies have demonstrated altered serum and tissue lipid profiles in CRC patients, characterized by increased very-long-chain fatty acids (VLCFAs) and reduced long-chain fatty acids (LCFAs), potentially driven by elongation of very-long-chain fatty acid (ELOVL) enzymes (Pakiet et al., 2019; Kondo et al., 2011). Lipidomic analyses have further shown that saturated triacylglycerols account for a substantial proportion of lipid perturbations during CRC progression, likely reflecting lipid droplet accumulation (Liu et al., 2019). Additional alterations, including decreased levels of glycolipids, glycerophosphocholine, and acylcarnitines, have also been reported in CRC serum samples (Tevini et al., 2022). Importantly, tissue-based lipidomic signatures, particularly triglyceride profiles, have emerged as independent prognostic markers capable of discriminating CRC subgroups according to mismatch repair status, oncogenic mutations (KRAS/BRAF), and tumour grade (Ecker et al., 2021).

Beyond biomarker discovery, lipidomics provides unique insights into lipid–protein, lipid–gene, and lipid–lipid interaction networks that underlie cancer biology. As dysregulated lipid metabolism is increasingly recognised as a hallmark of cancer (Snaebjornsson et al., 2020), clinical lipidomics has emerged as a translational subfield aiming to improve disease prediction, diagnosis, and therapeutic stratification (Dehairs et al., 2015; Moreno et al., 2022). Compared with traditional diagnostic approaches, lipidomics offers a systems-level view of metabolic dysregulation and enables precise identification of therapeutic targets and diagnostic biomarkers (Lv et al., 2018). Nonetheless, substantial challenges remain, including methodological heterogeneity, lack of standardization, and difficulties in cross-study comparison, which currently limit clinical translation. Addressing these challenges will be essential for integrating lipidomics into precision oncology and advancing its application in CRC management.

### 2.7 Epigenomics of CRC

Epigenetic dysregulation plays a central role in the initiation, progression, and therapeutic resistance of CRC, and epigenomic analyses have increasingly been applied to identify clinically relevant biomarkers and targets. Aberrant DNA methylation patterns have been associated with CRC screening, staging, and disease stratification. Tissue-based methylation markers such as SFMBT2, ITGA4, THBD, and ZNF304 have shown potential for early CRC detection, whereas methylation of KCNJ12, VAV3-AS1, and EVC has been linked to disease staging. In addition, NEUROD1 and FAM72C have been proposed as dual biomarkers to distinguish non-advanced precancerous lesions from inflammatory bowel disease (Zhang X. et al., 2020). Fecal DNA methylation markers, including SEPT9 and SDC2, have demonstrated value as non-invasive tools for early CRC detection (Zhao et al., 2020). These findings highlight the translational relevance of epigenetic alterations in CRC diagnosis and risk stratification.

Beyond DNA methylation, multiple layers of epigenetic regulation contribute to CRC pathogenesis. Histone modifications are increasingly recognised as key determinants of malignant phenotypes. For example, ubiquitination-mediated degradation of HDAC3 reduces histone acetylation and promotes cancer stem cell–associated gene expression, revealing a novel mechanism of epigenetic regulation in CRC (Zhan et al., 2020). Moreover, VprBP kinase–mediated phosphorylation of histone H2A at threonine 120 drives oncogenic transcriptional programs and enhances colon cancer cell proliferation, identifying VprBP as a potential therapeutic target (Ghate et al., 2021). Advances in epigenomic profiling technologies—including bisulfite sequencing for DNA methylation (Singer, 2019), chromatin immunoprecipitation sequencing (ChIP-seq) for histone modifications (Neganova et al., 2022), and multiple high-throughput methods for RNA modifications such as N6-methyladenosine (m6A) mapping (Dominissini et al., 2012; Linder et al., 2015; Molinie et al., 2016; Meyer, 2019; Zhang Z. et al., 2019; Wang Y. et al., 2020)—have enabled comprehensive characterization of epigenetic landscapes in CRC.

Despite these advances, substantial challenges remain. CRC exhibits pronounced intertumour and intratumour heterogeneity, with distinct genomic and epigenetic profiles across patients and even within individual tumours, complicating the identification of universally applicable biomarkers or therapeutic targets. Furthermore, most epigenomic studies have focused on coding regions, whereas the roles of non-coding elements—including regulatory regions and long non-coding RNAs—remain incompletely understood in CRC pathogenesis. Importantly, unlike genetic mutations, epigenetic alterations are potentially reversible, making them attractive targets for therapeutic intervention (Jung et al., 2020). The growing interest in epigenetic therapies, such as DNA methyltransferase inhibitors, reflects this therapeutic promise and underscores the need for deeper mechanistic insight into how epigenetic and genetic factors interact to drive CRC development and metastasis (Muller and Gyorffy, 2022; Rezapour et al., 2019; Thomas and Marcato, 2018). To provide a more clinically relevant overview of the major omics layers in CRC, Table 1 summarises representative biomarkers, sample types, clinical applications, and the translational evidence already described in the current literature.

**TABLE 1:** Clinical applications of multi-omics technologies in CRC.

Omics layer	Representative sample types	Key biomarkers/signatures	Main clinical applications	Representative clinical/translational evidence already described in the manuscript	Major limitations
Genomics	Tumour tissue, plasma cfDNA, ctDNA	KRAS, BRAF, PIK3CA, APC mutations; MSI status	Treatment selection, prognosis stratification, immunotherapy eligibility	Genomic profiling is described as a cornerstone of CRC management; MSI is described as a key biomarker that predicts PD-1 inhibitor efficacy in metastatic CRC	Limited functional insight; mutations do not always translate into phenotypic changes
Epigenomics	Tumour tissue, stool DNA, cfDNA	DNA methylation panels (e.g., SEPT9, SDC2, SFMBT2, KCNJ12)	Early screening, disease staging, non-invasive diagnosis	Tissue-based methylation markers are described as having potential for early detection and staging; stool SEPT9 and SDC2 are described as having demonstrated value as non-invasive early detection tools	Tumour heterogeneity; lack of standardized validation pipelines
Transcriptomics	Bulk tumour tissue, scRNA-seq	CMS subtypes; immune-related gene signatures; ncRNAs	Molecular subtyping, tumour microenvironment profiling, prognosis	scRNA-seq is described as revealing tumour microenvironment features and clinically relevant biomarkers; RNA-seq is described as enabling risk score models for prognosis	Batch effects; incomplete mRNA–protein concordance
Proteomics	Tumour tissue, plasma, serum	Protein panels (EGFR, LRG1, SAA2, ITIH4); PTMs	Early detection, risk stratification, therapy response assessment	Multiple blood-based protein panels are described as showing sensitivities exceeding 70% and specificities approaching 90%; several proteins are described as repeatedly validated in plasma and serum cohorts	Technical variability; limited generalisability across platforms
Metabolomics	Serum, plasma, urine, stool	Polyamines, bile acids, SCFAs, amino acid signatures	Early detection, disease monitoring, metabolic phenotyping	Metabolite profiles are described as distinguishing healthy individuals, benign polyps, and CRC, and as differentiating early-stage from advanced-stage disease	High biological variability; complex data interpretation
Lipidomics	Serum, tumour tissue	VLCFA/LCFA imbalance; glycerophospholipids; sphingolipids	Prognosis assessment, metabolic vulnerability identification	Tissue-based lipidomic signatures, particularly triglyceride profiles, are described as independent prognostic markers that discriminate CRC subgroups by mismatch repair status, KRAS/BRAF mutations, and tumour grade	Methodological heterogeneity; limited cohort validation
Microbiomics	Stool, tumour tissue	*Fusobacterium nucleatum*, microbial metabolite profiles	Screening, prognosis, treatment response prediction	Specific microbial taxa are described as enriched in adenoma and CRC and detectable in faecal samples; microbiome–metabolome signatures are described as having diagnostic potential	Causality unclear; inter-individual variability

## 3 Artificial intelligence (AI) in CRC

### 3.1 AI methodological framework for CRC multimodal analysis

AI methodologies used in CRC research can be understood across three complementary dimensions: learning paradigm, model architecture, and interpretability. At the broadest level, AI refers to computational approaches that support data-driven prediction and decision-making (Koteluk et al., 2021). Machine learning, a major subfield of AI, enables algorithms to identify patterns from large datasets and apply learned rules to new data for classification or prediction (Lapuente-Santana et al., 2021; Langley, 2011). Deep learning represents a further subset of ML based on multilayer neural networks, which can learn hierarchical feature representations directly from high-dimensional inputs (Hamet and Tremblay, 2017; Bengio, 2009).

From the perspective of learning paradigm, supervised learning remains the dominant strategy in clinically oriented CRC research because it relies on labelled data and is well suited to diagnostic, prognostic, and treatment-prediction tasks (Zhu et al., 2020; Alizadehsani et al., 2021; Bilal et al., 2021). Common supervised algorithms include logistic regression, support vector machine, naïve Bayes, gradient boosting, decision trees, random forests, and neural networks. Unsupervised learning, by contrast, identifies latent structure in unlabelled data and is mainly used for clustering, dimensionality reduction, and exploratory analyses (Hamet and Tremblay, 2017; Guo et al., 2023). Semi-supervised learning extends this framework by combining labelled and unlabelled data, thereby reducing annotation burden while improving model performance. Closely related weakly supervised approaches are particularly relevant to CRC digital pathology, where slide-level labels are often more feasible than exhaustive patch-level annotation. Reinforcement learning represents a distinct paradigm in which learning is guided by rewards or penalties linked to expected outcomes (Zhu et al., 2020; Alizadehsani et al., 2021).

Model selection in CRC is also shaped by data modality. Classical ML algorithms are most commonly applied to structured clinical variables, complete blood count indices, serum or stool biomarkers, and selected omics features after feature engineering. Their main advantages include lower data requirements, greater transparency, and easier integration with conventional clinical prediction frameworks. By contrast, DL models are most widely used for image-rich tasks, particularly in endoscopy, radiology, digital pathology, and surgical video analysis. Among these, convolutional neural networks (CNNs) have become central to image classification, detection, and segmentation, whereas recurrent neural networks (RNNs) and related sequential architectures have been explored for temporally ordered data. Compared with handcrafted-feature pipelines, DL approaches often achieve stronger predictive performance, but they typically require larger annotated datasets and are more vulnerable to limited interpretability, domain shift, and centre-specific artefacts (Hamet and Tremblay, 2017).

Recent methodological development has increasingly focused on reducing dependence on manual labels. Self-supervised learning enables models to learn transferable and discriminative feature representations from unlabelled data using internal learning signals rather than expert annotations (Niehues et al., 2023; Claudio Quiros et al., 2024). This shift has helped to establish the conceptual basis for foundation models, which are large-scale pretrained models that can be adapted to multiple downstream tasks with limited fine-tuning (Chen et al., 2024; Wang et al., 2024). Such approaches are particularly promising for CRC pathology and multimodal prediction because they may improve transferability across tasks and cohorts while lowering annotation requirements.

Recent advances in general-purpose foundation models and large multimodal models have further extended this paradigm. Medical variants of the Gemini family, including Med-Gemini, are based on multimodal architectures capable of processing and reasoning across text, images, and code, thereby supporting more complex tasks such as clinical reasoning, literature synthesis, report generation, and early diagnostic assistance (Alhur, 2024; Sonoda et al., 2024; Yang et al., 2024; Wagner et al., 2020; Fedus et al., 2022). In computational pathology, models such as CHIEF, RetCCL, and CTransPath illustrate how large-scale pretraining can improve transferability across diverse downstream tasks. CHIEF, developed through unsupervised tile-level pretraining and weakly supervised whole-slide pretraining on large-scale histopathology data, has shown strong generalisability across centres, scanners, and specimen types, and has demonstrated robust performance in tumour origin classification, genomic profile prediction, MSI prediction in CRC, and survival stratification (van der Laak et al., 2021; Song et al., 2023; Fu et al., 2020; Kather et al., 2020; Echle et al., 2021; Wagner et al., 2023; Azizi et al., 2023). RetCCL further highlights the value of clustering-guided contrastive learning for whole-slide representation learning and interpretable image retrieval without task-specific fine-tuning (Wang et al., 2023). Likewise, CTransPath combines a convolutional neural network backbone with a multi-scale Swin Transformer and uses semantically relevant contrastive learning to derive transferable histopathology representations across multiple downstream tasks, including weakly supervised whole-slide classification and colorectal adenocarcinoma gland segmentation (Wang et al., 2022). In radiology, Merlin represents a 3D vision-language foundation model for CT imaging that was pretrained using paired CT scans, radiology reports, and structured electronic health record data, with both internal and external validation across a broad range of clinically relevant tasks. Collectively, these models suggest that large-scale pretraining, self-supervised or weakly supervised learning, and multimodal alignment may improve feature reuse, generalisation, and label efficiency across pathology and radiology tasks relevant to CRC.

PROGPATH, CRCFound, and DINOPath represent recent advances in foundation AI models for cancer prognosis and personalized treatment. PROGPATH integrates histopathological image features with routinely collected clinical variables to achieve pancancer survival prediction using a weakly supervised deep learning framework. Trained on 7,999 whole-slide images from 6,670 patients across 15 cancer types and validated on 7,374 images from 4,441 patients in 17 external cohorts, it demonstrated robust performance across cancer types, stages, treatment modalities, and biomarker-defined subgroups, while interpretability analyses identified key morphological patterns driving risk stratification (Yuan et al., 2025). CRCFound, a self-supervised CT image foundation model for colorectal cancer pretrained on 5,137 unlabelled scans, achieved strong adaptability and generalisation across six diagnostic and two prognostic tasks, addressing the challenge of limited annotated data (Yang et al., 2025). DINOPath, trained on over 130 million patches from 104,876 whole-slide images of gastrointestinal cancers, accurately predicted disease-free and disease-specific survival, stratified patients into clinically relevant risk groups, and predicted benefit from adjuvant chemotherapy, demonstrating the potential for foundation models to complement clinicopathologic factors and inform personalized therapy (Wang X. et al., 2025). Collectively, these models highlight the promise of large-scale, self-supervised, and multimodal AI for robust, generalizable, and clinically actionable cancer prognosis and treatment planning.

Interpretability represents a separate but equally important dimension. In principle, AI models can be viewed as explainable, partially explainable, or black-box systems according to the extent to which their internal decision process can be understood by human experts (Keyl et al., 2025; Binder et al., 2021; Shephard et al., 2024). Explainable models aim to provide insight into the features or patterns driving prediction, whereas black-box models—often highly complex architectures—may achieve strong performance at the cost of limited transparency (Coudray et al., 2018). Accordingly, the methodological value of an AI system in CRC should not be judged by predictive accuracy alone, but also by its suitability for the input modality, annotation burden, interpretability requirements, and potential for robust clinical translation.

### 3.2 Applications in CRC screening

Screening is essential for reducing CRC incidence and mortality by enabling early detection and timely intervention (Siegel et al., 2021). Although colonoscopy remains the most effective screening tool (Issa and Noureddine, 2017), its performance is limited by operator dependence and a substantial miss rate, particularly for small or flat lesions (van Rijn et al., 2006). AI–assisted endoscopy has emerged as a promising solution, substantially improving the detection, classification, localisation, and segmentation of colorectal polyps while reducing inter-observer variability. Deep learning–based systems have demonstrated high sensitivity and specificity for real-time identification of diminutive and non-polypoid lesions, shortening reading time and reducing missed diagnoses (Yamada et al., 2019; Park et al., 2024; Wang J. et al., 2025).

Beyond endoscopy, AI-driven risk stratification models using large-scale population data have expanded CRC screening strategies. Machine learning approaches integrating demographic variables (Hilsden et al., 2018), complete blood count indices (Hornbrook et al., 2017; Kinar et al., 2016), serum or stool biomarkers (Ivancic et al., 2020; Pan et al., 2021), and multi-omics data have shown robust predictive performance (Nazari et al., 2021; Wan et al., 2019). In primary care settings, CBC-based models achieved AUCs above 0.80 and significantly increased case detection when combined with FOBT, supporting their role as scalable triage tools for colonoscopy referral (Park et al., 2024; Hornbrook et al., 2017; Kinar et al., 2016). In parallel, blood-based protein and N-glycan biomarkers analysed with machine learning have demonstrated high sensitivity for detecting advanced adenomas and CRC (Ivancic et al., 2020; Pan et al., 2021), while plasma cfDNA sequencing combined with ML enabled accurate detection of early-stage CRC (AUC up to 0.92) (Wan et al., 2019).

Mismatch repair deficiency (dMMR) and MSI are critical biomarkers in CRC, present in approximately 15% of cases and closely linked to immunotherapy response (De’Angelis et al., 2018; Vilar and Gruber, 2010; Saillard et al., 2023). Recent studies have shown that deep learning models can directly predict MSI status from histopathological images, achieving strong discriminatory performance without the need for immunohistochemistry or genetic testing (Jiang et al., 2022). More recent work has extended this field from proof-of-concept prediction towards benchmarking of pathology foundation models for MSI assessment in CRC, highlighting the importance of external generalisability and model comparison across architectures (Bilal et al., 2026). AI-based tools such as MSIntuit further support clinical MSI screening by reliably excluding microsatellite-stable tumours and accurately identifying MSI cases (Saillard et al., 2023). Nevertheless, challenges remain, including limited generalisability, lack of standardised thresholds across laboratories, and insufficient long-term outcome data. Future CRC screening strategies should therefore focus on integrating AI technologies into evidence-based pathways while carefully balancing early detection benefits against the risks of overdiagnosis (Mori et al., 2021; Ozawa et al., 2020).

### 3.3 Applications in CRC diagnosis and staging

The diagnosis of CRC encompasses both qualitative confirmation and disease staging, aiming to establish pathological evidence of malignancy and to assess tumour extent and severity (Song et al., 2019). Conventional qualitative diagnosis relies on biopsy obtained during endoscopy or surgery followed by histopathological evaluation, whereas staging primarily depends on radiological imaging, including CT and MRI. The integration of AI into diagnostic workflows seeks to enhance efficiency, reduce clinician workload, improve image interpretability, and ultimately decrease rates of misdiagnosis and missed diagnosis.

Histopathology remains the gold standard for CRC diagnosis and underpins postoperative staging (Acs et al., 2020), yet diagnostic accuracy is affected by inter- and intra-observer variability. AI applications in digital pathology have therefore focused on gland segmentation and tumour classification. Deep learning–based models have demonstrated robust performance in segmenting colorectal glands and distinguishing benign from malignant tissues, achieving high accuracy and reproducibility across heterogeneous datasets (Ding et al., 2020; Yuan et al., 2024). Machine learning classifiers, including support vector machines and hybrid feature-based approaches, have further improved CRC detection and grading accuracy, in some cases exceeding 95% (Yuan et al., 2024; Rathore et al., 2015). In addition, AI-assisted analysis of immunohistochemistry images has shown promise in predicting lymph node metastasis, suggesting potential complementary roles to conventional histopathology (Takamatsu et al., 2019). Likewise, multi-institutional clinical validation studies have begun to extend digital pathology AI towards more specific staging-related tasks, including automated detection of lymph node metastasis in CRC specimens (Giammanco et al., 2024). Emerging studies combining pathological images with immune and genetic data further support AI-enabled individualized diagnostic strategies relevant to targeted therapy and immunotherapy (Ge et al., 2019).

Radiological imaging constitutes the cornerstone of CRC staging, and AI-driven radiomics has become a major focus of diagnostic research (Yun and Guangwei, 2020). CT-based computer-aided detection systems have demonstrated high sensitivity for identifying flat or subtle lesions, supporting their role as non-invasive screening and diagnostic adjuncts (Taylor et al., 2008). Convolutional neural network–based models applied to CT have also shown performance comparable to expert radiologists in detecting hepatic metastases, while offering improved diagnostic confidence (Khalili et al., 2020). Notably, radiomic approaches have enabled the non-invasive prediction of molecular features such as KRAS mutation status directly from CT images, providing a potential alternative to invasive tissue sampling (González-Castro et al., 2020). More recent original studies continue to support the feasibility of CT-based radiomics for non-invasive KRAS prediction in CRC, reinforcing the translational potential of radiogenomic modelling (Wang W. et al., 2025). In rectal cancer, deep learning applied to MRI has achieved high accuracy in tumour and lymph node segmentation and metastasis prediction, with markedly reduced interpretation time compared with radiologists (Lu et al., 2018; Trebeschi et al., 2017).

Despite these advances, most AI applications in CRC diagnosis remain focused on pathological and radiological images (Acs et al., 2020; Bad et al., 2021). Challenges include limited interpretability of deep learning “black-box” models (Zhao and Hastie, 2019), variability in image acquisition quality, lack of standardized public datasets, and insufficient integration of clinical data. Moreover, AI-driven diagnostic tools linked to targeted therapies and immunotherapy remain underexplored, and robust prospective validation is still lacking. Nevertheless, AI-assisted diagnostic systems have shown promising gains in selected CRC tasks, particularly in digital pathology and radiology, but reported performance remains dependent on image quality, task definition, and validation setting, and robust prospective evidence is still limited (Mori et al., 2018; Wang P. et al., 2020; Liang et al., 2016).

### 3.4 Applications in CRC treatment

Treatment of CRC relies on chemotherapy, surgery, and multidisciplinary comprehensive management strategies (Bondeven et al., 2015). The integration of AI into CRC treatment has primarily focused on enhancing treatment safety, optimizing surgical management, and supporting clinical decision-making.

One important clinical application of AI in systemic therapy is the management of treatment-related toxicity. Irinotecan (CPT-11), a commonly used chemotherapeutic agent for CRC, is associated with substantial adverse effects that often limit its clinical use. Machine learning models incorporating clinical variables and serum biomarkers have been developed to predict irinotecan-induced toxicities, including diarrhea, leukopenia, and neutropenia, thereby assisting clinicians in individualized risk assessment and treatment planning (Oyaga-Iriarte et al., 2019). In contrast, AI-based toxicity management strategies for other standard CRC agents remain underexplored.

AI has demonstrated particularly strong potential in CRC surgery. Deep learning–based systems have been developed for automatic recognition of surgical phases and actions during laparoscopic and robotic colorectal procedures, achieving high accuracy and enabling real-time analysis of surgical workflows (Kitaguchi et al., 2020; Kitaguchi et al., 2022; Kolbinger et al., 2024). Image-based AI models have further been applied to identify anatomical structures and tissue planes, supporting intraoperative guidance and surgical standardization (Igaki et al., 2022). In addition, AI-assisted analysis of intraoperative perfusion imaging and perioperative clinical data has shown promise in predicting surgical complications, such as anastomotic leakage, thereby contributing to risk stratification and perioperative decision-making (Park et al., 2020; Mazaki et al., 2021).

Beyond intraoperative applications, AI technologies have also been applied to surgical training and postoperative management. Deep learning models trained on large-scale surgical video datasets can objectively assess surgical skills and procedural quality, offering valuable tools for colorectal surgical education (Kitaguchi et al., 2021). Moreover, machine learning models integrating perioperative clinical data have been developed to predict postoperative outcomes, including length of hospital stay, readmission, and short-term mortality, supporting postoperative care planning and healthcare resource optimization (Masum et al., 2022).

Overall, although AI applications in CRC treatment remain at an early stage and are limited by small datasets and lack of prospective validation (Huang et al., 2020), current evidence suggests that AI may improve selected aspects of treatment-related risk estimation, surgical workflow analysis, and perioperative management; however, the clinical magnitude of benefit has not yet been established through prospective evaluation. Future efforts should prioritize multimodal data integration, standardized model development, and real-world clinical validation to facilitate the translation of AI technologies into routine CRC treatment pathways.

### 3.5 Applications in CRC prognosis

Prognosis in CRC mainly involves predicting recurrence and long-term survival (Huang et al., 2020). Traditional prognostic models based on clinicopathological variables and conventional statistics are limited in their ability to capture the biological heterogeneity and complex, nonlinear interactions that drive disease progression. AI offers a data-driven framework capable of integrating multidimensional information and extracting high-order features, thereby enabling more accurate and individualized prognostic assessment.

AI-based models have been widely applied to recurrence prediction following curative resection, which is critical for postoperative surveillance and adjuvant treatment selection (Weiser et al., 2008). Early nomogram-based approaches improved risk stratification beyond standard staging systems but remained constrained by intrastage heterogeneity. More recent machine learning and deep learning methods have demonstrated superior performance in stage-specific recurrence prediction, particularly in stage II and IV CRC, by leveraging clinical variables and imaging-derived features (Takenaka et al., 2020; Xu Y. et al., 2020; Li et al., 2019). These approaches allow for finer risk discrimination and may help identify patients most likely to benefit from intensified follow-up or additional therapy.

Survival prediction represents another major focus of AI-driven CRC prognosis. Deep learning models applied to digitized haematoxylin–eosin–stained pathology slides have shown that prognostic information can be directly extracted from tumour histomorphology, achieving expert-level performance in outcome prediction (Kather et al., 2019). In addition, AI-based quantification of spatial immune features in whole-slide images has yielded clinically relevant prognostic biomarkers, with CD3CT emerging as a stage-independent predictor of overall survival in CRC (Cai et al., 2024). Recent multimodal studies suggest that combining histopathology with additional clinical and imaging information may further improve prognostic stratification beyond single-modality pathology models (Shi et al., 2025). For example, PRISM-CRC integrated histopathology, radiology, endoscopy, and clinical data within a deep learning framework, achieving a concordance index of 0.82 for 5-year disease-free survival prediction and an AUC of 0.91 for microsatellite instability classification, while also showing that performance may decline under domain shift (Shi et al., 2025). In parallel, AI models using structured clinical data have modestly but consistently outperformed clinician-based prognostication in estimating long-term survival (Sailer et al., 2015). More biologically informed multimodal approaches have further linked radiological phenotypes to RNA sequencing profiles and histopathological patterns, revealing immune and stromal features associated with differential benefit from adjuvant chemotherapy in stage II CRC (Xie et al., 2025). Tumour microenvironment-related information has also been incorporated into multimodal frameworks to improve prediction of both survival and chemotherapy benefit in large CRC cohorts (Ji et al., 2025). Taken together, these studies suggest that multimodal prognostic frameworks may offer stronger predictive performance and finer risk stratification than single-modality models, although differences in robustness, interpretability, and implementation complexity remain important. Despite these advances, most studies remain retrospective, and challenges related to model interpretability, generalisability, and prospective validation continue to limit clinical translation (Kinar et al., 2016; Tai et al., 2018).

### 3.6 The combination of AI and multi-omics in CRC

In oncology, the rapid development of AI and big data analytics has enabled large-scale analysis of multi-omics datasets, thereby accelerating precision cancer research (Zafari et al., 2023). In CRC, AI-driven platforms have demonstrated the capacity to link molecular features with clinical phenotypes. For example, the MOMA platform can infer genetic alterations and predict prognosis directly from histopathological patterns (Tsai et al., 2023). Machine-learning models integrating genomics, transcriptomics, proteomics, and metabolomics data have generally shown better predictive discrimination than single-modality approaches in retrospective CRC studies, although the magnitude of improvement varies across cohorts, endpoints, and integration framework (Zhang S. L. et al., 2023; Tong et al., 2020; Yang et al., 2022). In addition, single-cell transcriptomic analyses have identified clinically relevant biomarkers (Zhang G. L. et al., 2019), while serum metabolomics and spatial proteomics have enabled robust patient stratification and biomarker discovery in CRC cohorts (Troisi et al., 2022; Levy et al., 2023).

Despite these advances, substantial challenges remain in applying AI to multi-omics data integration. The high dimensionality, heterogeneity, and scale of multi-omics datasets increase the risk of noise, overfitting, and reduced model interpretability if irrelevant features are not adequately controlled. Feature selection and dimensionality reduction techniques are therefore essential to enhance model performance. Moreover, differences in data types, distributions, and sample sizes across omics layers complicate integration and often require data transformation or mapping before downstream analysis. Additional challenges include class imbalance and missing data, which can be addressed through sampling strategies, cost-sensitive learning, or imputation methods such as random forests and k-nearest neighbors (Picard et al., 2021).

To address these issues, three principal strategies for multi-omics integration have been proposed: early, intermediate, and late integration. Early integration concatenates features from multiple omics layers but is prone to the curse of dimensionality, whereas late integration analyzes each omics layer independently before combining results. Intermediate integration, by contrast, projects heterogeneous data into shared or complementary latent representations, enabling more effective capture of cross-omics interactions and biological complexity (Cai et al., 2022). Within this framework, AI and machine-learning approaches have been widely applied to overcome data heterogeneity, dimensionality, missing values, scalability, and class imbalance (Mirza et al., 2019). Importantly, AI-based integration of multi-omics data with non-omics information such as clinical variables, laboratory tests, imaging, pathology, and electronic health records offers a powerful means to bridge genotype and phenotype.

However, the translational value of these models depends not only on predictive performance, but also on data quality, computational infrastructure, and robustness beyond the development setting. These requirements are particularly difficult to meet in CRC multi-omics research, where matched datasets are often limited in size, incomplete across modalities, and vulnerable to batch effects or cohort-specific bias (Abadir et al., 2020; Auger et al., 2020). Variability in clinical presentation, treatment pathways, and endpoint definition may further reduce model stability when algorithms are applied outside the conditions under which they were trained (Auger et al., 2020; Chen et al., 2019). Interpretability also remains a major challenge. Classical machine-learning models based on structured clinical or omics variables may offer greater transparency, whereas deep learning and multimodal fusion models can capture more complex nonlinear interactions at the cost of reduced interpretability. In such settings, the “black-box” problem becomes particularly important, because apparently strong performance may obscure unrecognised confounding, technically driven artefacts, or biologically implausible associations (Auger et al., 2020; Narla et al., 2018). Collectively, these considerations suggest that AI-driven multi-omics integration is highly promising for precision oncology in CRC, but its clinical value will depend on reproducibility, biological plausibility, and robust external validation. To compare AI applications across the CRC clinical continuum more critically, Table 2 summarises the major data modalities, analytical methods, representative performance reported in the manuscript, and the main validation or translational limitations currently highlighted in the field.

**TABLE 2:** AI applications across the CRC clinical continuum.

Clinical stage	Data modality	AI methodology	Representative application	Representative performance reported in the manuscript	Validation/key limitation described in the manuscript
Screening	Endoscopy images	CNN, deep learning	Real-time detection and classification of diminutive and non-polypoid colorectal polyps	High sensitivity and specificity for real-time identification; reduced missed diagnoses	Limited generalisability and insufficient long-term outcome data are described as ongoing challenges for AI screening applications
Screening	Blood/stool biomarkers	Machine learning (LR, RF, SVM)	Risk stratification using CBC indices, serum proteins, and stool markers	CBC-based models achieved AUCs above 0.80; blood-based protein and N-glycan biomarkers showed high sensitivity for advanced adenomas and CRC	Prospective validation not reported in the cited studies beyond the need for evidence-based integration into screening pathways
Screening	Plasma cfDNA	ML-based sequencing models	Early-stage CRC detection using cfDNA fragmentation and mutation patterns	AUC up to 0.92 for early-stage CRC detection	Prospective validation not reported in the cited studies
Diagnosis	Pathology whole-slide images	CNN, DL	Gland segmentation, tumour classification, MSI/dMMR status prediction	High accuracy and reproducibility across heterogeneous datasets; some ML classifiers exceeded 95%; MSI prediction showed strong discriminatory performance	Limited interpretability, lack of standardized public datasets, and insufficient robust prospective validation
Staging	CT/MRI imaging	Radiomics, DL	Lymph node metastasis prediction; tumour stage assessment	High accuracy for tumour and lymph node segmentation/metastasis prediction; reduced interpretation time compared with radiologists	Variability in image acquisition quality; robust prospective validation still lacking
Molecular profiling	Radiology images	ML-based radiogenomics	Non-invasive prediction of molecular features such as KRAS mutation status	Promising non-invasive molecular prediction described in current text	Prospective validation not reported in the cited studies
Treatment (surgery)	Surgical video streams	CNN, DL	Surgical phase recognition, anatomical structure identification	High accuracy for automatic recognition of surgical phases and actions; real-time workflow analysis	AI treatment applications are described as early stage, limited by small datasets and lack of prospective validation
Prognosis	Pathology + clinical data	ML, DL survival models	Recurrence risk stratification and survival prediction	Deep learning on H&E slides achieved expert-level performance; structured clinical-data models modestly but consistently outperformed clinician-based prognostication	Most studies remain retrospective; interpretability, generalisability, and prospective validation remain limiting factors
Integration	Multi-omics + clinical data	ML, DNN	Precision stratification and personalised clinical decision support	Multi-omics models improved accuracy for CRC outcomes and survival compared with single-modality approaches	High dimensionality, heterogeneity, missing data, and class imbalance complicate integration

## 4 Outlook and challenges

The integration of multi-omics technologies has substantially accelerated biomarker discovery in CRC by enabling systematic interrogation of molecular alterations across multiple biological layers. However, elucidating the mechanisms underlying biomarker generation and establishing the diagnostic and therapeutic relevance of candidate biomarkers remain challenging and are both critical for drug development. Integrative strategies that combine genomics, proteomics, and metabolomics have shown promise in improving tumour characterisation and supporting anticancer drug development, particularly with the continued advancement of high-throughput sequencing technologies (Eylem et al., 2020; Nam et al., 2021; Correa-Aguila et al., 2022).

Despite this potential, several barriers continue to limit the clinical translation of multi-omics discoveries. Omics approaches remain costly and technically demanding, while data quality is often compromised by non-standardised sampling, heterogeneous experimental protocols, and limited cohort sizes. In particular, metabolomics faces substantial analytical challenges because of the complexity and dynamic variability of metabolite profiles. Moreover, the absence of standardised evaluation frameworks and large-scale validation cohorts hampers reliable assessment of biomarker sensitivity and specificity. Tumour heterogeneity further complicates biomarker validation, highlighting the need for higher-resolution approaches, including single-cell and spatially resolved analyses.

The convergence of AI and multi-omics has reshaped cancer research, yet reliable translation still requires rigorous data standardisation, harmonisation, and quality control. Differences in data types, scales, and preprocessing pipelines across omics platforms may introduce bias or information loss during integration. Advanced computational strategies are therefore needed to handle dimensionality, missingness, and cross-platform heterogeneity, including feature selection, data augmentation, and biologically informed modelling (Amann et al., 2020; Grinsztajn et al., 2022; Dash et al., 2022). Improved data accessibility and sharing are also essential, as limited access to high-quality multi-omics datasets continues to constrain progress, despite established resources such as TCGA and GTEx.

Interpretability, dataset bias, and fairness represent additional and closely linked challenges. Many high-performing deep learning systems lack transparency, limiting clinical trust and impeding routine adoption (Amann et al., 2020). Although approaches such as tree-based models, SHAP-based explanations, and domain knowledge-guided modelling may improve interpretability (Grinsztajn et al., 2022; Dash et al., 2022), their practical value depends on whether such explanations support reliable human judgement rather than superficial reassurance. Recent work has increasingly emphasised both explainability and causability, arguing that AI systems should not only generate accurate outputs but also provide explanations that enhance human understanding and support actionable clinical decisions (Holzinger et al., 2019). This issue is particularly relevant in complex diagnostic contexts, such as histopathology, where clinicians require interpretable and biologically coherent evidence to justify clinical action.

Bias in multi-omics datasets may further impair model transportability and ethical acceptability. Differences in sequencing platforms, proteomic workflows, image acquisition quality, pathology staining, preprocessing pipelines, and cohort composition may cause models to learn centre-specific or platform-specific signals rather than disease biology. Incomplete multimodal datasets may also introduce selection bias when analyses are restricted to patients with complete data, while imputation strategies may propagate hidden uncertainty if not carefully validated (Picard et al., 2021). Underrepresentation of non-European populations in current datasets raises additional concern that AI systems may perform unevenly across demographic groups, thereby exacerbating existing inequities in precision oncology (Carter et al., 2020; Murdoch, 2021; Peterson et al., 2019; Martin et al., 2019). These limitations are not merely technical; they directly affect clinical reliability, fairness, and the safe deployment of AI across heterogeneous real-world populations.

**FIGURE 2:**
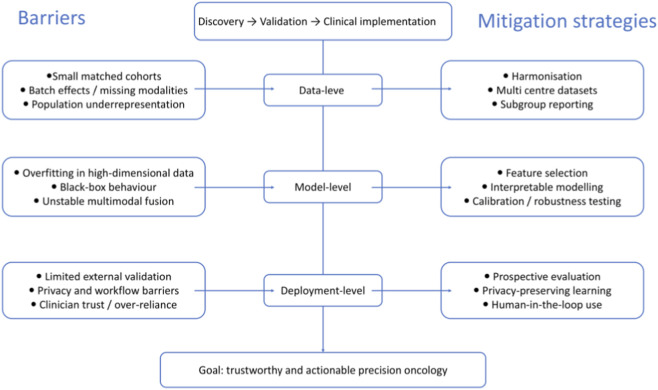
Barriers and mitigation strategies for translating AI-enabled multi-omics into precision oncology for colorectal cancer. Schematic overview of the major barriers and corresponding mitigation strategies affecting the translation of AI-enabled multi-omics in colorectal cancer (CRC) from discovery to validation and clinical implementation. At the data level, small matched cohorts, batch effects, missing modalities, and population underrepresentation limit data quality, representativeness, and generalisability; these challenges may be mitigated through data harmonisation, multi-centre datasets, and subgroup reporting. At the model level, overfitting in high-dimensional data, black-box behaviour, and unstable multimodal fusion reduce interpretability and robustness; potential solutions include feature selection, interpretable modelling, and calibration or robustness testing. At the deployment level, limited external validation, privacy and workflow barriers, and clinician mistrust or over-reliance hinder real-world adoption; corresponding strategies include prospective evaluation, privacy-preserving learning, and human-in-the-loop use. Together, these measures support the development of trustworthy and actionable precision oncology in CRC.

Rigorous validation remains essential before widespread implementation. Most current AI studies in CRC, including many models for prognosis and treatment prediction, remain retrospective and show substantial variability in reported sensitivity, specificity, and overall accuracy (Kinar et al., 2016; Huang et al., 2020; Tai et al., 2018; Wang et al., 2019). Broader deployment will therefore require validation beyond the originating centres and, where feasible, prospective or randomised evaluation to determine whether AI-assisted decision-making confers genuine clinical benefit (Abadir et al., 2020; Plana et al., 2022). In parallel, privacy protection must remain a central priority, particularly as academic, clinical, and commercial data ecosystems become increasingly interconnected (Tom et al., 2020). Privacy-preserving frameworks, including federated learning, swarm learning, and other distributed strategies, may support responsible data sharing and model development (Warnat-Herresthal et al., 2021; Cao et al., 2022), but such approaches must still be accompanied by transparent reporting, subgroup performance assessment, and careful regulatory oversight. Finally, trust should be regarded as an outcome of evidence rather than an assumption. Although explainable AI is often proposed as a solution to the black-box problem, poorly calibrated explanations may paradoxically increase blind reliance on algorithmic outputs and reduce critical clinical appraisal (Jacobs et al., 2021; Cabitza et al., 2023). Thus, future progress in AI-driven multi-omics for CRC will depend not only on more powerful algorithms, but also on prospective validation, transparent reporting, biologically plausible interpretation, equitable dataset design, and clinically feasible implementation pathways (Reis-Filho and Kather, 2023). The major challenges and potential solutions are summarised in Figure 2.

## 5 Conclusion

CRC management increasingly depends on integrative molecular stratification, and multi-omics approaches have expanded the range of clinically relevant biomarkers and biological insights. However, the clinical translation of these data remains limited by heterogeneity, high dimensionality, and incomplete interpretability.

AI provides a powerful analytical framework to integrate multi-omics data with imaging and clinical information, facilitating early screening, accurate diagnosis, individualized treatment, and refined prognostic assessment. Emerging evidence suggests that AI-driven multi-omics integration may outperform single-modality approaches in selected CRC applications, particularly in risk stratification and prognosis modelling, although current evidence remains heterogeneous and is still dominated by retrospective studies.

Despite these advances, significant challenges remain, including data standardisation, model generalisability, interpretability, and ethical considerations related to privacy and fairness. Addressing these issues will require multidisciplinary collaboration and robust clinical validation. Ultimately, the convergence of AI and multi-omics is poised to transform CRC management by bridging molecular complexity with actionable clinical decision-making.
